# Molecular regulatory mechanisms of *Populus deltoides* under combined high temperature and short-photoperiod stress associated with DNA methylation

**DOI:** 10.48130/forres-0026-0025

**Published:** 2026-07-31

**Authors:** Zheng-sai Yuan, Wei-xi Zhang, Xiao-hua Su, Zheng-hong Li, Jing Zhang, Lu-lan Miao, Hang Wei, Zi-hao Wang, Yan-guang Chu, Chang-jun Ding

**Affiliations:** 1 State Key Laboratory of Tree Genetics and Breeding, Research Institute of Forestry, Chinese Academy of Forestry, Beijing 100091, China; 2 Binhai Forestry Research Center of National Forestry and Grassland Administration, Research Institute of Forestry, Chinese Academy of Forestry, Beijing 100091, China; 3 Co-Innovation Center for Sustainable Forestry in Southern China, Nanjing Forestry University, Nanjing 210037, China

**Keywords:** *Populus deltoides*, DNA methylation, Combined environmental stress, Adaptability, High temperature and short photoperiod

## Abstract

*Populus deltoides* is a key species for industrial timber and ecological construction in temperate regions, where increasingly frequent and persistent heat waves pose serious challenges to its survival. However, the epigenetic mechanisms by which DNA methylation regulates environmental responses remain poorly understood. Here, whole-genome bisulfite sequencing and RNA-seq were performed on five *P. deltoides* genotypes grown in temperate and tropical regions. Results revealed that CG/CHG methylation stability is closely correlated with environmental sensitivity. Significant CG/CHG methylation variations may occur specifically in sensitive genotypes with large provenance-environment differences, thereby threatening the survival of *P. deltoides* by inhibiting the expression of key genes involved in life processes. CHH methylation variation may act as a potential epigenetic regulator of environmental adaptation. Promoter CHH-hypermethylation appears to represent a general response of *P. deltoides* under high-temperature and short-photoperiod (HS) stress, potentially regulating the expression of 56 genes to activate Ca^2+^ influx and heat shock proteins, repressing auxin, cytokinin, and cell cycle pathways, thereby initiating stress-protective responses. *PdeCNGC13*, *PdeARF6*, *PdeLOG3,* and *PdeHSP15.7* were identified as potential regulatory genes of HS adaptation that are associated with DNA methylation. The mechanism of *PdeLOG3* may involve HS-induced CHH-hypermethylation at its *PdeLOG3* promoter, which may be associated with suppressed expression, reduced dihydrozeatin levels, and growth. This effect was partially reversed by 5-Azacytidine administration, accompanying increased cytokinin synthesis, enhanced antioxidant capacity, and coinciding with alleviation of HS stress. Our work preliminarily reveals the molecular mechanisms underlying DNA methylation-associated environmental adaptation in poplar, providing potential genetic targets for breeding climate-resilient trees.

## Introduction

Plant habitats are undergoing unprecedented and intense changes, particularly due to the increasing frequency and persistence of global heat waves. These conditions pose severe survival challenges for sessile plants that cannot relocate to more favorable environments^[1−3]^. The stress imposed on plants by high temperatures is not an isolated phenomenon. Instead, it disrupts the equilibrium of light energy distribution within the plant. This disruption occurs through competition for energy and interference with signaling processes, ultimately disrupting the delicate balance between temperature and light^[3,4]^. Studies have shown that temperature and light are critical factors shaping plant introduction, domestication, and adaptive capacity. Acting both independently and synergistically, these physical cues influence plant responses to both gradual and sudden environmental fluctuations, thereby maintaining a dynamic balance between growth and stress resistance^[1,4−6]^.

As a key component of epigenetic regulation, genome-wide changes in DNA methylation are closely associated with the ability of plants to adapt to climatic, biotic, and abiotic stresses^[7,8]^. Evidence indicates that DNA methylation enhances the adaptability of *Arabidopsis thaliana*, *Oryza sativa*, *Cucumis sativus*, and *Fragaria vesca* to stresses such as salinity, drought, temperature, and light by modulating the expression of genes involved in signaling, hormone biosynthesis, and stress response^[7−12]^. For instance, CHH-type methylation variation in *C. sativus* mediates high-temperature responses by altering the expression of genes linked to reactive oxygen species (ROS) metabolism and auxin biosynthesis pathways^[13]^. Moreover, stress-sensitive genotypes, such as thermosensitive lines, typically display higher levels of DNA methylation than stress-insensitive genotypes^[7,12,14]^.

The adaptive regulation of DNA methylation under environmental stress varies according to sequence context and functional region^[3,7]^. CHH-type methylation has been widely implicated in regulating plant responses to temperature and light^[7,10,12]^. In *A. thaliana*, CHH-type methylation of transposons increases with temperature, a variation strongly associated with the DNA methyltransferase CMT2^[7]^. By contrast, in *O. sativa*, CHH-type methylation levels are suppressed under favorable light conditions^[12]^. Unlike CHH-type methylation, CG- and CHG-type modifications are regarded as more conserved and less susceptible to environmental factors. However, some studies have found that CG/CHG methylation plays a more prominent role in regulating plant adaptation to climate. For instance, differences in CG/CHG-type methylation levels are more strongly correlated with the climate of provenance, particularly latitude and temperature, than with the conditions of the planting site^[5,7]^. *Populus deltoides* is a key species for industrial timber and ecological construction in temperate regions, where the increasing frequency and persistence of heat waves pose significant challenges to its growth and survival. To date, studies in *Populus* have mainly reported methylation changes associated with stress responses or developmental transitions under small-scale geographic shifts or under controlled conditions^[15,16]^. However, little is known about the sequence contexts, functional regions, biological processes, and key genes through which DNA methylation regulates the environmental adaptability of *Populus* under large-scale geographic and climatic variation, particularly under combined temperature and light stress. Therefore, it is essential to investigate DNA methylation dynamics and their regulatory mechanisms in *P. deltoides* exposed to combined environmental stress, with the goal of supporting cross-regional introduction, domestication, and improved climate resilience.

In this study, we performed comprehensive analysis of whole-genome bisulfite sequencing (WGBS) and RNA-seq on five *P. deltoides* genotypes grown in temperate and tropical regions, complemented by climate-chamber experiments with DNA methylation inhibitor treatments. Field experiments showed that significant CG/CHG-type methylation variation occurred specifically in *P. deltoides* genotypes with large differences in provenance and planting environments, which may be associated with reduced survival of *P. deltoides*, potentially through the suppression of genes involved in fundamental physiological processes. In contrast, CHH-type promoter hypermethylation may represent a general response across genotypes of *P. deltoides* under HS stress, potentially influencing the expression of 56 genes that are associated with Ca^2+^ influx and heat shock proteins (HSPs) while simultaneously correlating with the repression of auxin and cytokinin pathways, which may contribute to stress-protective responses. The application of the DNA methylation inhibitor 5-Azacytidine demonstrated that *PdeCNGC13* (Ca^2+^ influx channel protein), *PdeARF6* (auxin response factor), *PdeLOG3* (cytokinin biosynthesis gene), and *PdeHSP15.7* (heat shock protein) are potential methylation-regulated effector genes for HS adaptation. Moreover, we propose a potential epigenetic pathway for *PdeLOG3* wherein HS-induced CHH-hypermethylation of the *PdeLOG3* promoter may correlate with the suppression of its transcription, which is in turn associated with reduced dihydrozeatin levels and growth. This effect appeared to be partially reversed by 5-Azacytidine administration, potentially enhancing HS tolerance. These findings preliminarily uncover the molecular mechanisms underlying DNA methylation-associated environmental adaptation in poplar, and provide potential genetic targets for breeding climate-resilient trees.

## Materials and methods

### Plant materials and growth conditions

**Figure 1:**
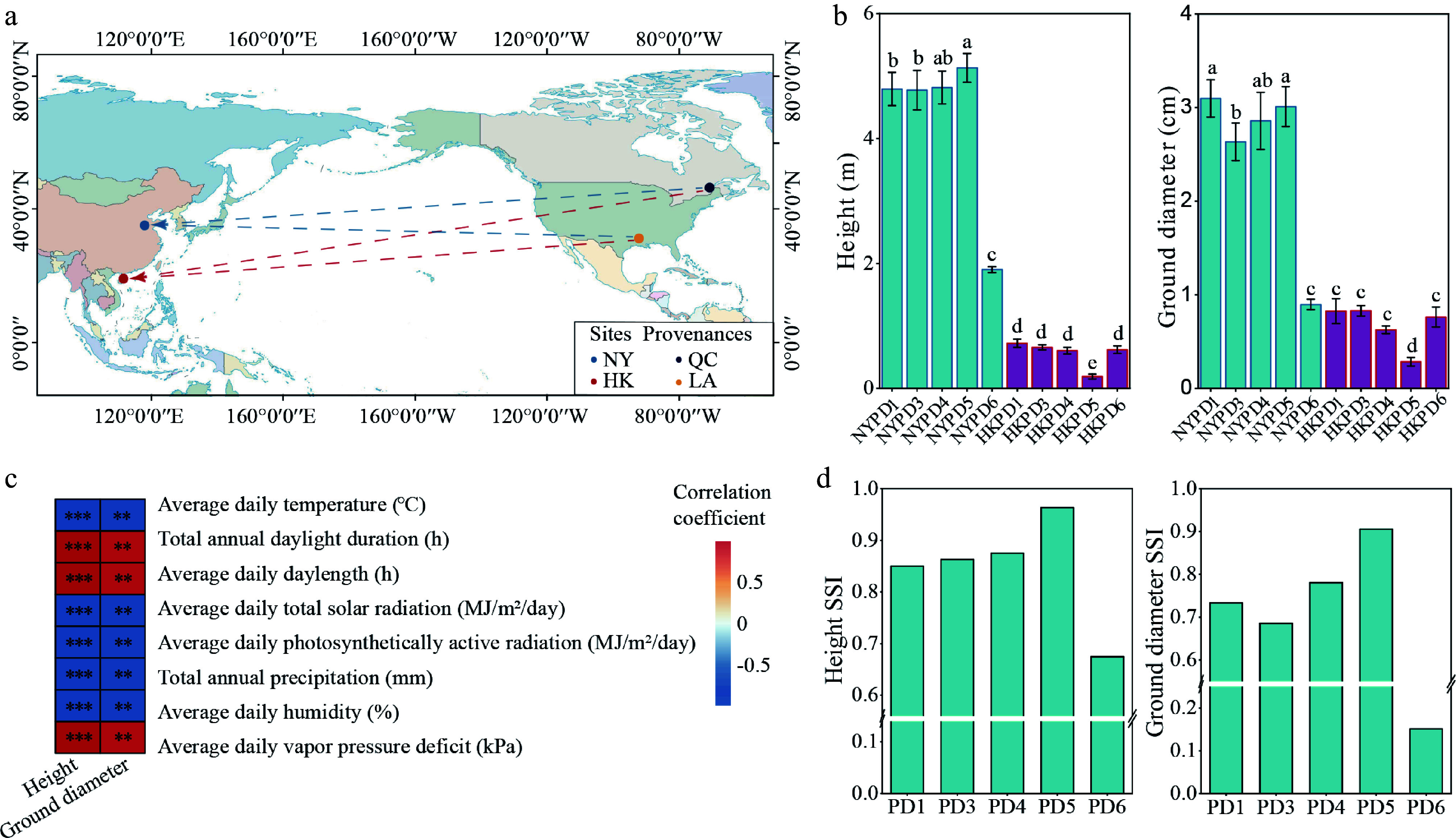
Differences in growth and SSI of *P. deltoides* cultivated between tropical and temperate monsoon regions. (a) Distribution of provenances and planting locations for five *P. deltoides* genotypes. Provenances include QC for PD5 and LA for PD1/3/4/6. Planting locations include HK, in the tropical monsoon climate zone, and NY, in the warm temperate monsoon climate zone. Both QC and LA are geographically closer to NY in terms of latitude. The base map was sourced from the National Standard Map Service System (http://bzdt.ch.mnr.gov.cn/), under the approval number GS (2016) 1663. (b) Net growth in tree height and ground diameter. Multiple comparisons were conducted using the Tukey test (*n* ≥ 6), with different lowercase letters indicating significant differences (*p* < 0.05). (c) Heatmap of correlations between net growth in tree height and ground diameter and climatic factors. The climate data are the average value from 2019 to 2021. Pearson's correlation, ** *p* < 0.01, *** *p* < 0.001. (d) SSI values for tree height and ground diameter. Error bars represent ± SD. QC, Quebec; LA, Louisiana; HK, Dingan, Haikou, Hainan; NY, Ningyang, Taian, Shandong; SSI, stress susceptibility index.

From the 14 *P. deltoides* genotypes previously identified by our group as exhibiting superior growth performance in at least one of Dingan, Haikou, Hainan (HK, 19°41′ N, 110°19′ E) and Ningyang, Taian, Shandong (NY, 35°46′ N, 116°48′ E)^[17]^, five were selected for further analysis in the present study. These included PD1, PD3, PD4, and PD6, originally introduced from Louisiana (LA), USA, and PD5 from Quebec (QC), Canada. To minimize the influence of provenance background, a germplasm bank was established in Ningyang, Taian, Shandong (35°55′ N, 116°53′ E) in 2009. This germplasm bank was geographically close to the subsequent temperate monsoon climate site, allowing all genotypes to undergo long-term domestication and propagation under local conditions. Cuttings collected from this germplasm bank were propagated in the greenhouse of the Chinese Academy of Forestry (40°0′ N, 116°14′ E) until April 2019, when uniformly grown seedlings were simultaneously transplanted to two distinct climatic regions: a tropical monsoon climate site in HK and a temperate monsoon climate site in NY (Fig. 1a). Detailed descriptions of the germplasm collection, introduction, and regional adaptation trials have been reported in our previous studies^[6,17]^, and detailed climate data for the two sites are provided in Supplementary Table S1. Both experimental forests were established using a randomized block design with three replicates and three plots per replicate, at a spacing of 0.6 m × 0.6 m. Tree height and ground diameter were recorded for each genotype from April 2019 to May 2021 to calculate net growth. The stress susceptibility index (SSI) was calculated as 1 - (Y_HK/Y_NY), where Y_HK and Y_NY represent the net growth of a genotype in the HK and NY locations, respectively. A higher SSI value indicates greater sensitivity to the environmental change from NY to HK, and poorer adaptability. In May 2021, leaves were sampled from both the south- and north-facing sides of each plant at one-third of the tree height. Equal amounts of leaves were pooled, immediately frozen in liquid nitrogen, and stored at –80 °C. Each genotype at each site was represented by three biological replicates, resulting in a total of 30 samples.

### Whole-genome bisulfite sequencing (WGBS)

Genomic DNA was extracted from *P. deltoides* leaves using the cetyltrimethylammonium bromide (CTAB) method. DNA integrity and contamination were assessed by agarose gel electrophoresis, while purity and concentration were determined with a NanoPhotometer spectrophotometer (Implen, Cupertino, CA, USA) and a Qubit DNA Assay Kit (Life Technologies, Carlsbad, CA, USA), respectively. High-quality DNA was randomly sheared into 200–300 bp fragments using a Covaris S220 ultrasonicator (Covaris, Woburn, MA, USA) and subjected to bisulfite conversion with an EZ DNA Methylation-Gold™ Kit (Zymo Research, Irvine, CA, USA). Library construction was carried out by Novogene Corporation (Beijing, China). The quality of the libraries was evaluated with a Bioanalyzer 2100 system (Agilent Technologies, Shanghai, China), and sequencing was performed on an Illumina NovaSeq platform (Illumina, San Diego, CA, USA).

### DNA methylation data analysis

The quality of raw reads was assessed using FastQC (v0.11.5; www.bioinformatics.babraham.ac.uk/projects/fastqc/), and low-quality or contaminated sequences were filtered with fastp (v0.20.0)^[18]^ to generate clean reads. Clean reads were aligned to the *P. deltoides* reference genome (https://phytozome-next.jgi.doe.gov/info/PdeltoidesWV94_v2_1) using Bismark for methylcytosine (mC) detection^[19]^. A binomial distribution test was applied to each cytosine site to confirm true mC, with thresholds set at sequencing depth ≥ 5 and *q*-value ≤ 0.01^[20]^. The methylation level at each site was calculated as mC/(mC + umC). Differentially methylated regions (DMRs) were identified using DSS^[21]^, with a minimum length of 50 bp, containing at least three cytosine sites, and > 50% of sites showing significant differences (determined by a Wald test, *p* < 1e-05). Adjacent DMRs separated by < 100 bp were merged into a single region. The minimum differential methylation level used to define DMRs was set at 0.1 for CG, CHG, and CHH sequence contexts. The identified DMRs were annotated using the *P. deltoides* reference genome. Promoter regions were defined as the 2 kb region upstream of the transcription start site (TSS). The TSS and transcription end site (TES) regions were defined as a ±1 kb window centered on the annotated start and end sites, respectively. Exon, intron, and transposable element (TE) region coordinates were derived from the genome annotation file and RepeatMasker analysis. For overlapping annotations, features were assigned priority in the following order: Promoter > TSS > TES > Exon > Intron > TE.

### Transcriptome sequencing and data analysis

RNA was extracted from the same batch of *P. deltoides* leaves used for WGBS with TRIzol Reagent (Thermo Fisher Scientific, Waltham, MA, USA). RNA-seq libraries were prepared using the NEBNext Ultra RNA Library Prep Kit for Illumina (New England Biolabs, Ipswich, MA, USA)^[22]^. Library quality was evaluated using a Bioanalyzer 2100 system (Agilent Technologies, Santa Clara, CA, USA), and sequencing was performed on an Illumina NovaSeq platform (Illumina, San Diego, CA, USA). Raw reads were quality-filtered with fastp (v0.19.7)^[18]^ to generate clean reads, which were then aligned to the *P. deltoides* reference genome using HISAT2 (v2.0.5)^[23]^. Gene expression levels were quantified with featureCounts (v1.5.0) in the Subread package^[24]^. Differentially expressed genes (DEGs) were identified using DESeq2 (v1.20.0)^[25]^, with thresholds set at |log_2_(fold change)| ≥ 1 and adjusted *p*-value ≤ 0.05. KEGG functional enrichment analysis of DEGs was performed using the clusterProfiler R package^[26]^ against the KEGG database^[27]^, applying a significance threshold of *p* ≤ 0.05. Short time-series expression miner (STEM) trend analysis of gene expression data was performed using the OE Cloud platform (https://cloud.oebiotech.com). Prior to analysis, the data were normalized by calculating the log_2_(fold change) for all genes across all samples, using the average expression level of the control sample NYPD6 (genotype PD6 from the NY region) as the baseline reference.

### HS combined stress and 5-Azacytidine (5-AzaC) treatment

Based on correlation analyses between growth and climatic data from field experiments, high temperature and short photoperiod were inferred to be important environmental factors limiting poplar growth in the HK region. To simulate these differences, seedlings of PD4, PD5, and PD6 (45-day-old cuttings) were grown under three regimes: normal temperature and long daylight (NL; constant 22 °C, 16 h light/8 h dark), high temperature and long daylight (HL; 30 °C/26 °C day/night, 16 h light/8 h dark), and high temperature and short photoperiod (HS; 30 °C/26 °C day/night, 8 h light/16 h dark). Plants were treated with either 100 mL of 5-AzaC (75 μM) or distilled water (control) every 2 d for 14 d. Each treatment included three replicates, with three plants per replicate. The SSI of growth traits for each genotype under HL and HS stress was calculated as 1 - (Y_HL/Y_NL) and 1 - (Y_HS/Y_NL), where Y_NL represents the growth of a genotype under NL conditions, and Y_HL and Y_HS represent its growth under HL and HS stress, respectively. Growth conditions were maintained at a light intensity of 1,000 μmol·m^−2^·s^−1^ and relative humidity of 80%.

### Physiological and hormonal assays

After 7 d of stress treatment, root Ca^2+^ flow rates were measured using Non-invasive Micro-test Technology with iFluxes 1.0 software (YoungerUSA, LLC, Amherst, MA, USA). Measurements were performed in the elongation zone, 600 µm from the root tip, with a slope of 25–29 mV/decade, and recorded continuously for over 5 min. Each treatment included three biological replicates, with three root tips measured per replicate as technical replicates. The contents of malondialdehyde (MDA) and proline, and the activity of peroxidase (POD) were determined using commercial reagent kits (BC0025, BC0295, and BC0095; Solarbio, Beijing, China). Auxin, cytokinin, and abscisic acid contents were quantified by ultra-performance liquid chromatography (ExionLC™ AD, SCIEX, Concord, ON, Canada) coupled with tandem mass spectrometry (QTRAP®6500+, SCIEX). Chromatographic and mass spectrometric conditions followed established protocols^[28,29]^. Data acquisition and quantification were performed using Analyst 1.6.3 and MultiQuant 3.0.3 software (SCIEX). Hormone assays were carried out by Metware Biotechnology Co., Ltd (Wuhan, China).

### Quantitative RT-PCR (qRT-PCR)

Total RNA was extracted from each sample using the RNAprep Pure Plant Plus Kit (Tiangen, Beijing, China). First-strand cDNA was synthesized with the PrimeScript RT Kit (TaKaRa, Otsu, Japan). Gene-specific primers were designed using the Primer3Plus online platform (www.primer3plus.com). All qRT-PCR assays were performed with SYBR Premix Ex Taq II (Takara) on a LightCycler® 480 II Real Time PCR Instrument (Roche, Basel, Switzerland) according to the manufacturer's protocol. Relative gene expression levels were calculated using the 2^−ΔΔCᴛ^ method, with Actin serving as the internal control. The primers used for qRT-PCR are listed in Supplementary Table S2.

### Bisulfite-specific PCR (BS-PCR) and DNA methylation site detection

Genomic DNA was extracted from poplar leaves using the Hi-DNAsecure Plant Kit (Tiangen). Bisulfite conversion of DNA from each sample was performed with the DNA Bisulfite Conversion Kit (Tiangen). Primers targeting the *PdeLOG3* gene were designed using the MethPrimer online tool (www.urogene.org/cgi-bin/methprimer/methprimer.cgi). BS-PCR was conducted with EpiTaq™ HS (TaKaRa, for bisulfite-treated DNA). PCR products were purified using the TIANgel Midi Purification Kit (Tiangen) and sequenced by Sangon Biotech (Shanghai, China) using the Sanger method. Sequencing data were aligned and assembled with BioEdit software (v7.0.9.0). Because bisulfite treatment caused DNA fragmentation, two pairs of primers (m*PdeLOG3*-1F/R and m*PdeLOG3*-2F/R; Supplementary Table S2) were designed to amplify overlapping fragments of the *PdeLOG3* target region, which were subsequently spliced for analysis.

## Results

### Variation in growth and stress susceptibility of *P. deltoides* genotypes across temperate and tropical regions

The ongoing northward shift of climatic zones and contraction of suitable habitats under intensifying climate change pose a growing threat to *P. deltoides*, highlighting the urgent need to breed climate-resilient poplar cultivars and to elucidate the underlying adaptive mechanisms^[30]^. In a previous study, we conducted large-scale regionalization trials in both temperate (NY) and tropical (HK) regions^[17]^. Following two years of continuous growth, we identified five genotypes that not only survived in the tropical region but also exhibited improved growth performance in at least one of the two sites. Growth measurements revealed that both tree height and ground diameter of these five genotypes were significantly greater in NY than in HK, with values ranging from 3.07−27.25 and 1.18−10.57 times those in HK, respectively (Fig. 1a, b). To investigate the main environmental factors limiting poplar growth in tropical regions, we performed correlation analyses between net growth and major climatic factors. Correlation analysis showed that growth traits across the five genotypes were significantly positively correlated with day length and vapor pressure deficit (VPD) (Pearson test, *p* < 0.01), and significantly negatively correlated with temperature, solar radiation, photosynthetically active radiation (PAR), precipitation, and humidity (Fig. 1c), suggesting that the unique site conditions in the HK region, particularly high temperature and short photoperiod, may be associated with the growth inhibition of *P. deltoides* in this region. Further detailed analysis of genotype-specific growth differences between the two regions revealed that the five genotypes exhibited distinct growth responses to the contrasting environmental conditions of the two sites. Specifically, significant differences in tree height and ground diameter (Tukey test, *p* < 0.05) were observed between the two sites for PD1, PD3, PD4, and PD5, with PD5 showing the most pronounced reduction in growth, and PD6 exhibiting minimal difference (Fig. 1b). Accordingly, the SSI values for tree height and ground diameter were highest in PD5 (0.96 and 0.91), followed by PD1, PD3, and PD4 (0.85–0.87 and 0.69–0.78), and lowest in PD6 (0.67 and 0.15), respectively (Fig. 1d). Based on these observations, the five genotypes were grouped as follows: PD6 (insensitive to environmental stress in HK), PD1/3/4 (moderately sensitive), and PD5 (highly sensitive).

### DNA methylation patterns of different *P. deltoides* genotypes in temperate and tropical regions

**Figure 2:**
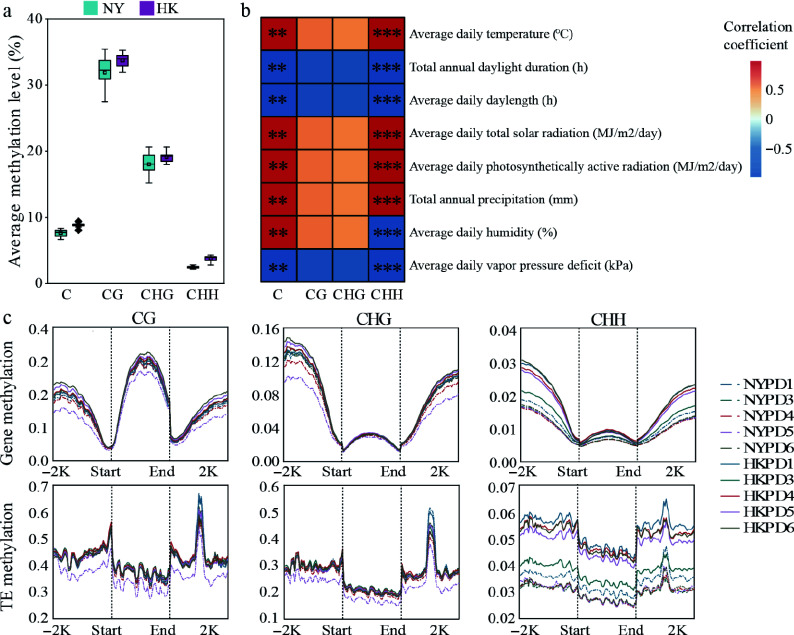
Differences in DNA methylation of *P. deltoides* cultivated between tropical and temperate monsoon regions. (a) Average methylation levels of all C sites, and C sites in CG, CHG, and CHH sequence contexts in the leaf genomes of different *P. deltoides* genotypes planted in NY and HK. For each genotype at each location, values represent the mean of three replicates, with five genotypes per location serving as replicates (*n* = 5). (b) Heatmap of correlations between average methylation levels in each sequence context and climatic factors. Pearson's correlation, ** *p* < 0.01, *** *p* < 0.001. (c) DNA methylation profiles of gene bodies, TEs, and their respective upstream 2 kb and downstream 2 kb regions, including those of mCG, mCHG, and mCHH. Error bars represent ± SD. HK, Dingan, Haikou, Hainan; NY, Ningyang, Taian, Shandong; TE, transposable element.

To explore whether these growth and sensitivity differences were associated with DNA methylation patterns, we performed WGBS on leaf samples from all five genotypes grown in both regions. In total, sequencing of the 30 samples yielded 1.668 billion raw reads and 1.631 billion clean reads, with 56.39%–75.92% uniquely mapped to the reference genome (*P. deltoides* WV94_v2_1). The average genome-wide coverage depth ranged from 13.85 to 24.16, and bisulfite conversion rates exceeded 99% in all samples (Supplementary Table S3), confirming high data quality suitable for subsequent analyses. Overall, the average methylation levels varied markedly across sequence contexts in *P. deltoides*, with CG context exhibiting the highest levels (27.47%–35.41%), followed by CHG (15.21%–20.61%), and the lowest in CHH (2.19%–4.30%; Fig. 2a).

Comparative analysis of methylation levels across different sites and *P. deltoides* genotypes revealed that the differences in DNA methylation levels between sites and among genotypes were also sequence-context specific. Relative to NY, HK samples exhibited higher average methylation levels, particularly in CHH (1.53-fold increase; Fig. 2a). Consistent with this, the correlation patterns of methylation profiles between samples varied across sequence contexts (Supplementary Fig. S1). For CG and CHG methylation, only the highly sensitive genotype PD5 showed apparent differences between the two locations. For the other four genotypes, methylation levels of the same genotype were similar across locations, suggesting that methylation in these sequence contexts may be primarily influenced by genotypic factors. In contrast, CHH methylation profiles were more similar within each location (i.e., within NY or within HK), particularly in HK. Furthermore, compared with mCG and mCHG, mCHH average methylation levels showed stronger correlations with climatic factors, exhibiting a significant positive correlation with temperature (Pearson test, *p* < 0.001) and a significant negative correlation with daylength (Fig. 2b). These findings suggest that CHH methylation levels may be predominantly influenced by environmental factors, and that the distinctive stressful environment in HK may have driven convergence in its methylation patterns.

Genotype-specific analysis showed that the highly sensitive genotype PD5 was unique; only NYPD5 displayed significantly reduced mCG and mCHG methylation levels in the 2 kb flanking regions of protein-coding genes and transposable elements (TEs) compared with HKPD5. This specificity may be related to the higher latitude of PD5 provenance (QC). In contrast, for mCHH methylation, levels in the 2-kb flanking regions of protein-coding genes and TEs were similar among genotypes but differed significantly between locations, with uniformly higher levels in HK for all genotypes (Fig. 2c). This highly consistent methylation variation across genotypes may be associated with the adaptive growth of *P. deltoides* in the HK region.

### CG/CHG and CHH methylation exhibit functional divergence in the response of *P. deltoides* to HK environmental stress

To investigate the potential causes underlying the differences in growth and sensitivity among genotypes between the two regions, we identified DMRs between genotypes within each location. In both regions, the majority of DMRs were CG-type (45,334–64,000 in NY; 53,353–85,126 in HK), followed by CHG-type (21,110–30,694 in NY; 22,293–45,764 in HK), while CHH-type DMRs were least abundant (3,564–12,094 in NY; 680–1,629 in HK) (Supplementary Fig. S2). In NY, the number of CG- and CHG-type DMRs was consistent across all genotype–genotype comparisons. By contrast, in HK, comparisons involving HKPD5 and the other four genotypes exhibited markedly higher numbers of DMRs (79,186–85,126 for CG; 43,091–45,764 for CHG) than other comparisons (53,353–57,744 for CG; 22,293–25,921 for CHG), primarily due to a higher proportion of hyper-DMRs (Supplementary Fig. S2a, S2b). Moreover, the number of CG- and CHG-type DMRs in PD5 vs other genotypes was greater in HK than in NY, whereas no such regional difference was observed for other genotypes, suggesting that the specific environmental conditions in HK may induce greater CG- and CHG-type methylation divergence in the sensitive genotype PD5. In contrast, CHH-type DMRs of five genotypes were significantly fewer in HK (680–1,629) than in NY (3,564–12,094), indicating that the environmental conditions in HK may promote convergence of CHH methylation patterns among genotypes (Supplementary Fig. S2c).

**Figure 3:**
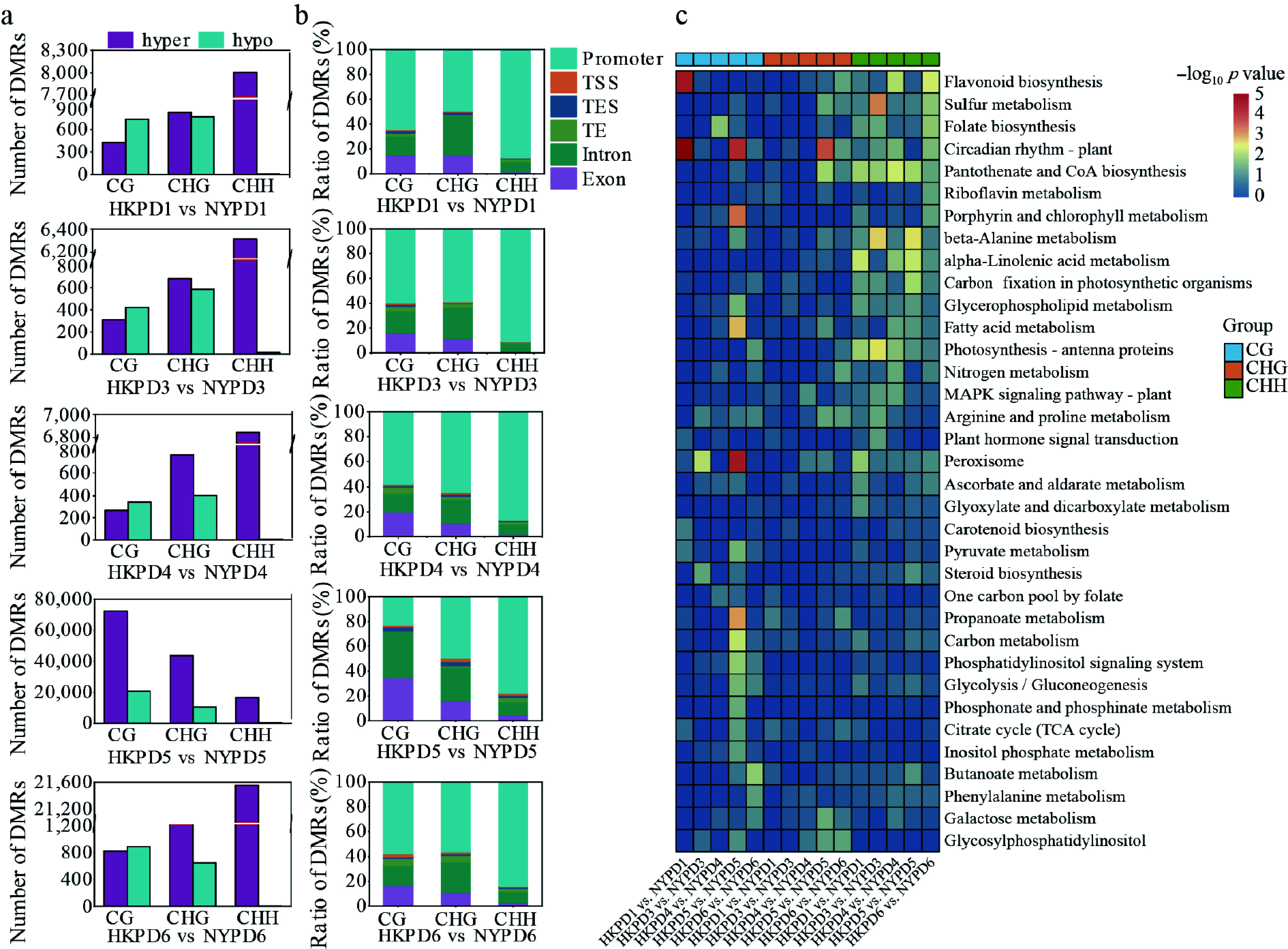
Identification of DMRs and functional annotation of corresponding DMGs between locations. (a) Number of DMRs in each comparison group. (b) Distribution of DMRs across gene functional regions, including promoter, intron, exon, TSS, TES, and TE, and their relative proportions in total DMRs. (c) Heatmap of KEGG enrichment analysis of CG-, CHG-, and CHH-type DMGs in each comparison group.

Next, DMRs between the same genotype grown in different climates (NY as control) were compared. The number of DMRs in HKPD5 vs NYPD5 was 6.20–19.67 times higher than in other comparisons, largely attributable to extensive CG- and CHG-type DMRs in promoter, intron, and exon regions (Fig. 3a, b). In HKPD5 vs NYPD5, CG- and CHG-type DMRs far outnumbered CHH-type DMRs, whereas in the other four genotypes, DMRs were predominantly CHH-type (Fig. 3a). Notably, CHH-DMRs did not vary with genotype sensitivity and were mainly hypermethylated in promoter regions, accounting for 76.84%–90.84% (Fig. 3a, b). Therefore, these hyper-promoter-CHH-DMRs may serve as the basis for a general stress response across genotypes to the HK environment. Supporting this, CHH-type differentially methylated genes (DMGs) shared among all five genotype comparisons (1,493) greatly exceeded unique ones (70–853), and nearly all were hypermethylated in promoter regions (Supplementary Fig. S3). By contrast, CG- and CHG-type DMGs were highly specific to HKPD5 vs NYPD5. KEGG enrichment analysis supported this functional distinction: CHH-DMGs were extensively and consistently enriched across all genotypes in pathways related to environmental stress responses, including redox processes, nitrogen metabolism, energy metabolism, and photosynthesis (Fig. 3c). By contrast, CG- and CHG-DMGs in HKPD5 vs NYPD5 were enriched in only a limited set of additional pathways, including carbon metabolism and the citric acid cycle, in a genotype-specific fashion (Fig. 3c). These results suggest a potential functional divergence between CHH-type and CG/CHG-type methylation in regulating growth and adaptability of *P. deltoides*. CHH methylation variation is closely associated with environmental factors and may contribute to the adaptability of *P. deltoides* to the HK environment, whereas CG/CHG methylation occurs specifically in the highly sensitive genotype PD5 and may be involved in genotype-specific sensitivity regulation.

### Genotype-specific CG/CHG hypermethylation correlates with its high sensitivity to the HK environment

DNA methylation regulates diverse biological activities in plants by modulating the expression of target genes^[7,12]^. To investigate the key biological processes associated with DNA methylation in contributing to genotype-specific sensitivity and general stress responses of *P. deltoides* to the HK environment, we performed RNA-seq on 30 leaf samples collected in parallel with WGBS. Expression profiles of 15 randomly selected genes were validated by qRT-PCR, and the results were highly consistent with RNA-seq data (Supplementary Fig. S4).

**Figure 4:**
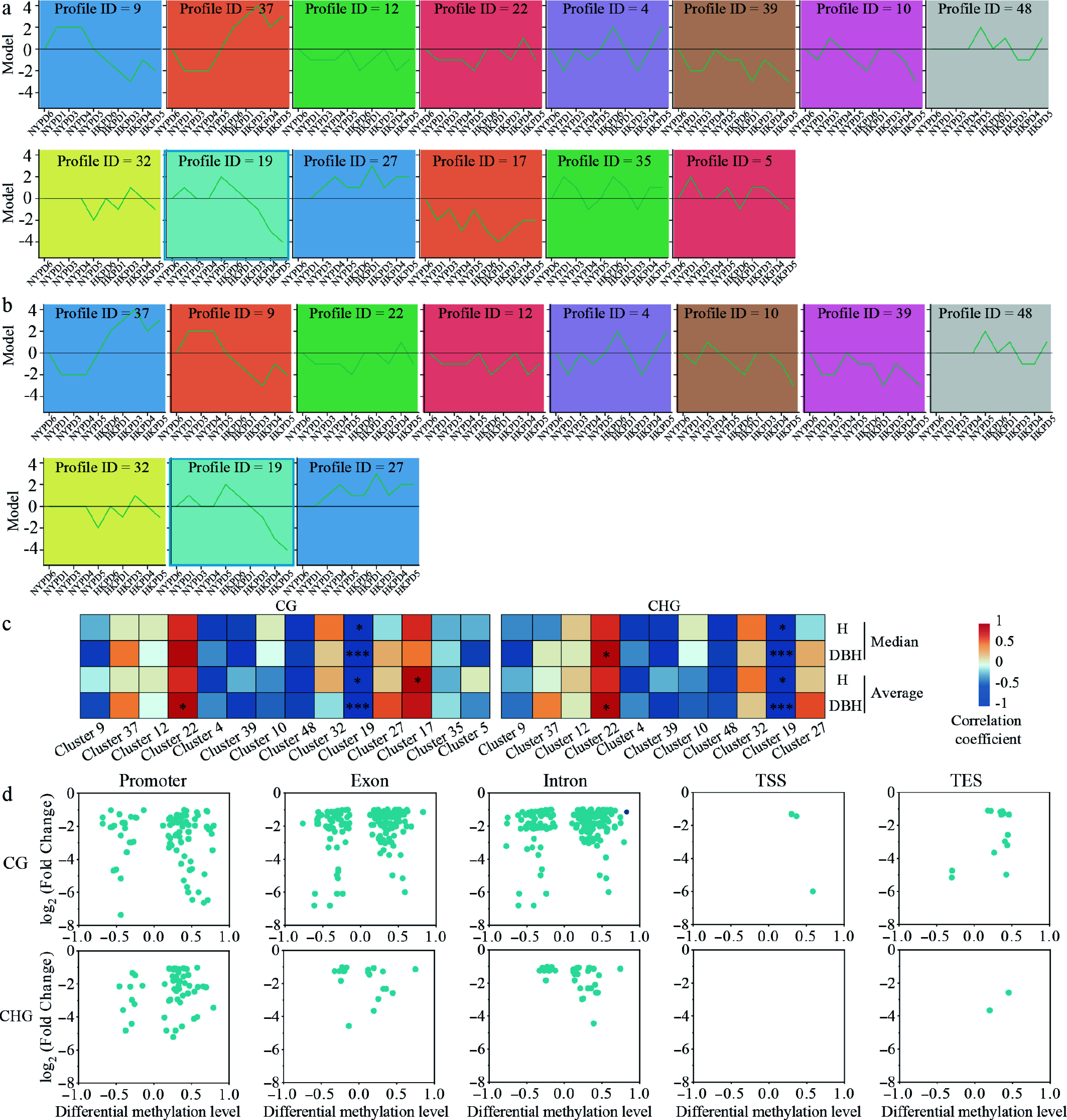
Identification of CG- and CHG-type DMGs associated with environment-sensitive genes and distribution of their DMRs across genic regions. STEM trend analysis of FPKM values for (a) CG-DMGs, and (b) CHG-DMGs specifically identified in HKPD5 vs NYPD5. For CG-DMGs and CHG-DMGs, 14 and 11 significant clusters were identified, respectively (FDR < 0.05). (c) The correlation between the expression trends of each cluster and the SSI of the genotypes. The expression trend of each cluster was primarily represented by the median of the normalized log_2_(fold change) values of its genes. The mean was also calculated as a robustness check, and both metrics yielded consistent results. Spearman's rank correlation was used for correlation analysis, and statistical significance was defined as * *p* < 0.05 and *** *p* < 0.001. CG-blue and CHG-blue clusters are highlighted with blue boxes. (d) Distribution of DNA methylation and gene expression differences in DMR–DEG pairs across functional regions of the CG-blue and CHG-blue clusters.

For genotype-specific sensitivity analysis, we performed STEM trend analysis on the 21,856 CG-DMGs and 9,740 CHG-DMGs specific to HKPD5 vs NYPD5, identifying two gene sets, designated CG-blue and CHG-blue, which were significantly negatively correlated with SSI (*p*-value < 0.05; Fig. 4a−c). CG-blue and CHG-blue contained 509 and 203 DMGs, respectively, with 148 and 59 genes significantly differentially expressed in HKPD5 vs NYPD5, and the magnitude of their expression differences was larger than those observed for other genotype–location comparisons. Notably, these genes were predominantly downregulated in HKPD5 (Supplementary Tables S4, S5). Consistent with genome-wide patterns, the majority of DMRs in CG-blue (148) and CHG-blue (59) occurred in promoter, exon, and intron regions (77–90 and 19–53, respectively), whereas relatively few were located near TSS or TES (3, 14; 0, 2) (Figs 3b, 4d). Under HK environment, CG- and CHG-type methylation in promoter, intron, and exon regions was predominantly hypermethylated and accompanied by lower expression (66.10%–73.04% for CG; 57.89%–81.13% for CHG), and the strongest effects were observed in promoter regions (73.04% and 81.13%) (Fig. 4d).

Further Functional enrichment analysis indicated that CG-blue genes were largely enriched in fundamental biological processes, including DNA replication, protein processing, carbon and sugar metabolism, and amino acid synthesis (Supplementary Fig. S5). In contrast, CHG-blue genes were enriched in only a limited number of pathways, such as glycolysis/gluconeogenesis. Therefore, we speculate that the high sensitivity and poor growth of genotype PD5 in tropical regions are associated with widespread CG/CHG hypermethylation in its promoter and genic regions, particularly CG. This epigenetic suppression may cripple the expression of genes essential for basic metabolic and biosynthetic processes, such as DNA replication, protein processing, and energy metabolism, potentially leading to an inadequate energy supply to mount an effective stress response.

### CHH-type promoter hypermethylation is associated with the general adaptive response of *P. deltoides* to the HK environment

**Figure 5:**
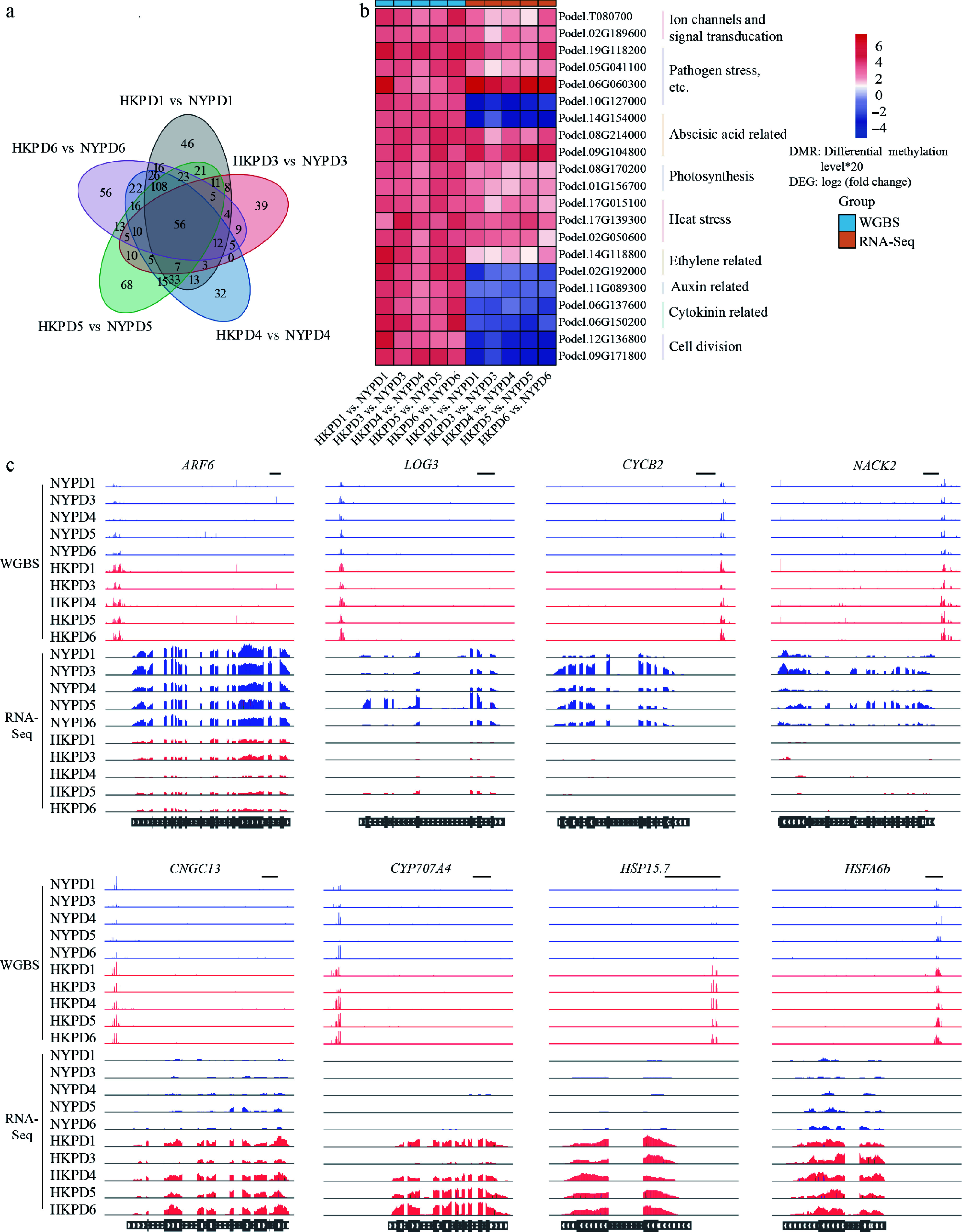
Identification of key CHH-methylated genes involved in stress adaptation of *P. deltoides* to the HK environment. (a) Venn diagram of hyper-promoter-CHH-DMDGs from five comparison groups. (b) Heatmap of DNA methylation and gene expression differences in key CO-hyper-promoter-CHH-DMDGs. Methylation differences represent the mean across multiple DMRs per gene. (c) IGV plots of promoter-CHH DNA methylation and gene expression levels in key CO-hyper-promoter-CHH-DMDGs. Bar, 500 bp.

To define the role of CHH-type methylation in contributing to the general response of *P. deltoides* to the HK environment, we analyzed 1,493 shared CHH-DMGs with hypermethylation in promoter regions identified across five genotype comparisons (same genotype grown in NY vs HK). Among these, 189–406 significantly differentially expressed DMGs (DMDGs) were detected per comparison group. Notably, the proportion of genotype-specific DMDGs (32–68; 8.82%–20.63%) was relatively low in all cases, including sensitive genotype PD5 (16.75%) (Fig. 5a). This suggests that a similar regulatory mechanism may exist across different genotypes. We identified 56 shared hyper-promoter-CHH-DMDGs (CO-hyper-promoter-CHH-DMDGs) that were consistently hypermethylated and differentially expressed in the same direction across all five comparisons (Supplementary Table S6). The expression of these key genes may be modulated by CHH-type hypermethylation in promoter regions in a functionally distinct manner. Specifically, genes involved in growth-promoting pathways, including auxin (*ARF6*), cytokinin (*LOG3*), and cell cycle regulation (*CYCB2* and *NACK2*), exhibited hypermethylation and reduced expression. In contrast, stress-response genes associated with Ca^2+^ influx (*CNGC13*), ABA catabolism (*CYP707A4*), heat shock response (*HSP15.7*, *HSFA6b*), and photoperiod sensing (*PRS1*) showed reduced expression (Supplementary Table S6; Fig. 5b, c). These findings suggest that the conserved CHH-type hypermethylation in promoter regions may contribute to transcriptional reprogramming in *P. deltoides* in response to environmental stress in HK. This epigenetic mechanism appears to simultaneously inhibit growth-related processes and enhance stress-protective responses, thereby fine-tuning the plant's overall acclimation to the environmental conditions in the HK region.

### *PdeLOG3* promoter demethylation correlates with enhanced HS tolerance via cytokinin and antioxidant pathways

Our results revealed that the five genotypes surviving under high-intensity stress in the HK region consistently exhibited CHH methylation variation, which was closely associated with high temperature and short photoperiod (Figs 1c, 2b). We therefore speculate that CHH-type methylation may play a potential role in contributing to the adaptation of *P. deltoides* to high temperature and short photoperiod conditions in tropical regions. To test this hypothesis, we examined CHH-type methylation and gene expression patterns, along with physiological traits, in PD4, PD5, and PD6 seedlings treated with 5-Azacytidine (5-AzaC) under NL, HL, and HS conditions in an artificial climate chamber.

**Figure 6:**
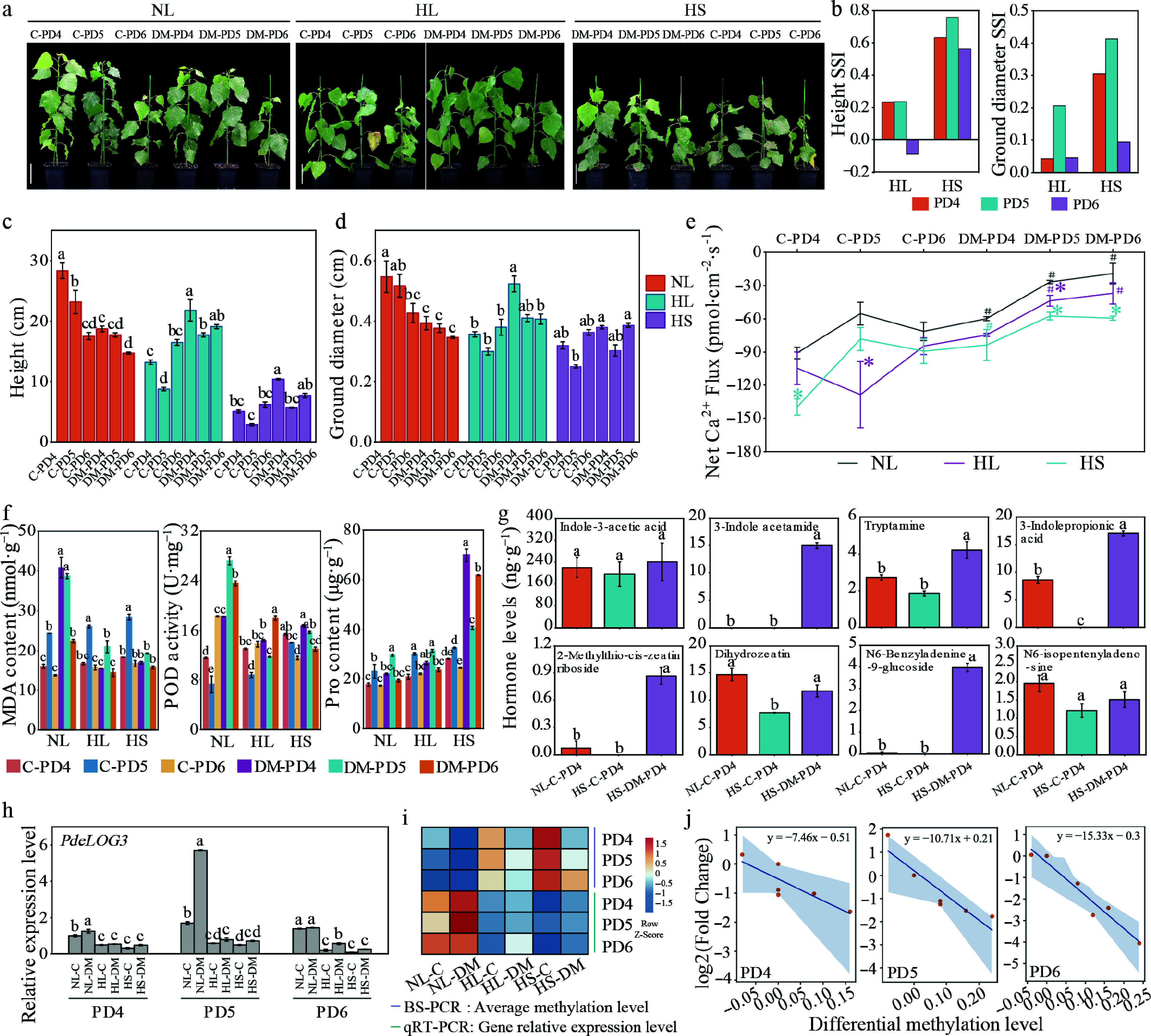
Phenotypic, physiological and molecular responses of *P. deltoides* to stress in the presence or absence of 5-AzaC. (a) Phenotypic characteristics under stress. Bar, 10 cm. (b) SSI for tree height and ground diameter. (c), (d) Net growth in tree height and ground diameter. (e) Ca^2+^ flow rate. * indicates that, under otherwise identical conditions, there was a significant difference between HL or HS and NL (*n* = 3, *p* < 0.05); purple denotes significance between NL and HL, and blue between NL and HS. # indicates a significant difference (*n* = 3, *p* < 0.05) between 5-AzaC-treated and untreated plants of the same genotype under identical conditions. (f) MDA content, POD activity, and proline content. (g) Auxin and cytokinin contents. (h) Relative expression level of *PdeLOG3*. (i) Methylation level and expression of the target fragment of *PdeLOG3*, with heatmap values representing the mean of four replicates. (j) Regression analysis of differential methylation level vs differential gene expression (log_2_ fold change) of the *PdeLOG3* target fragment, with NL-C as the control. Each point represents a treatment, shown as the mean of four replicates; confidence interval = 0.99. (a) Phenotypic traits and (c), (d) net growth data were measured after 14 d of stress, whereas (e)–(j) physiological, biochemical, and molecular data were collected after 7 d. Multiple comparisons were conducted using the Tukey test (*n* ≥ 3), with different letters indicating significant differences (*p* < 0.05). Error bars represent ± SD. NL, normal temperature with long daylight; HL, high temperature with long daylight; HS, high temperature with short photoperiod; C, without 5-AzaC; DM, with 5-AzaC; SSI, stress sensitivity index.

Consistent with field performance, PD4 and PD5 exhibited greater growth inhibition in tree height and ground diameter than PD6 under HL and HS conditions, with the SSI values for these traits consistently following the order PD5 > PD4 > PD6 across all genotypes, a trend that was particularly pronounced for ground diameter (Fig. 6a–d). These results suggest that climate chamber experiments manipulating temperature and photoperiod can effectively recapitulate, at least in part, the growth and sensitivity differences among poplar genotypes observed between HK and NY, supporting the view that high temperature and short photoperiod are important environmental factors constraining poplar growth in the HK region. In addition, the application of 5-AzaC alleviated growth inhibition under HL and HS, whereas it repressed growth under NL conditions, with significant effects observed in PD4 and PD5 (Tukey test, *p* < 0.05; Fig. 6c, d). Consistent with this, 5-AzaC treatment reversed the stress-induced Ca^2+^ influx (significant under HL) and malondialdehyde (MDA) accumulation (significant in PD5) following HL and HS stress, while increasing peroxidase (POD) activity and proline content (Fig. 6e, f). In contrast, under NL conditions, 5-AzaC treatment caused a significant increase in MDA (1.59–2.56-fold). This implies that 5-AzaC may mitigate HS stress in *P. deltoides* by boosting antioxidant capacity, but may induce cellular damage under normal growth conditions (Fig. 6f). Furthermore, hormone analysis showed that HS stress suppressed the synthesis of several auxins and cytokinins, but this suppression was partially reversed by 5-AzaC treatment. For instance, the content of indole-3-acetic acid increased by 1.12–1.23 times that of untreated HS plants (Fig. 6g).

To identify key genes involved in the HS stress response in *P. deltoides*, we conducted qRT-PCR analysis on the core hyper-promoter-CHH-DMDGs that were previously identified. Our results revealed that the stress-induced upregulation of *PdeCNGC13* and *PdeHSP15.7* was attenuated by 5-AzaC application (Supplementary Fig. S6a, S6b). Conversely, the expression of *PdeARF6* and *PdeLOG3*, which was suppressed by stress, increased after 5-AzaC treatment (Supplementary Fig. S6c; Fig. 6h). However, no consistent expression patterns were detected for *PdeHSFA6b*, *PdeCYCB2*, *PdeNACK2* and *PdeCYP707A4*, as well as ABA content in the 5-AzaC treatment experiment (Supplementary Figs S6d–S6f, S7), which may be partially attributable to the relatively mild stress conditions and 5-AzaC dosage applied.

Notably, *PdeLOG3* expression most closely aligned with the observed growth and stress phenotypes under HS and 5-AzaC treatment (Fig. 6a–h). This gene encodes an enzyme catalyzing the conversion of cytokinin nucleotides into active nucleobases such as dihydrozeatin^[31−33]^. In our study, dihydrozeatin synthesis was significantly inhibited under HS (47.56% reduction compared to NL), but significantly rescued by 5-AzaC treatment (51.50% increase compared to HS control) (Fig. 6g). Critically, WGBS data revealed that promoter-CHH-DMRs of *PdeLOG3* were highly conserved across all five genotypes, encompassing a 291-bp region with 31 C sites (four CG, two CHG, and 25 CHH) (Fig. 5c; Supplementary Fig. S8a). These findings suggest that *PdeLOG3* may be an important gene in the general adaptive response to HS stress in *P. deltoides*. The BS-PCR analysis of this gene across the three genotypes supported this hypothesis. HL, HS, and 5-AzaC treatments significantly altered the methylation level of the target fragment, with this variation showing a tendency to be conserved across genotypes and restricted to a few specific C sites (Supplementary Fig. S8b, S8c). CHH-type methylation levels in this fragment increased by 0.08–0.16 under HL and by 0.16–0.24 under HS compared to NL. Notably, following 5-AzaC treatment, the CHH methylation levels under the corresponding stress conditions decreased by 0.04–0.08 (HL) and 0.06–0.16 (HS) compared to the untreated controls (Supplementary Fig. S8b; Fig. 6i). Furthermore, a strong negative correlation was observed between CHH methylation in the *PdeLOG3* promoter and its expression level (regression analysis, confidence interval 0.99; Fig. 6j).

**Figure 7:**
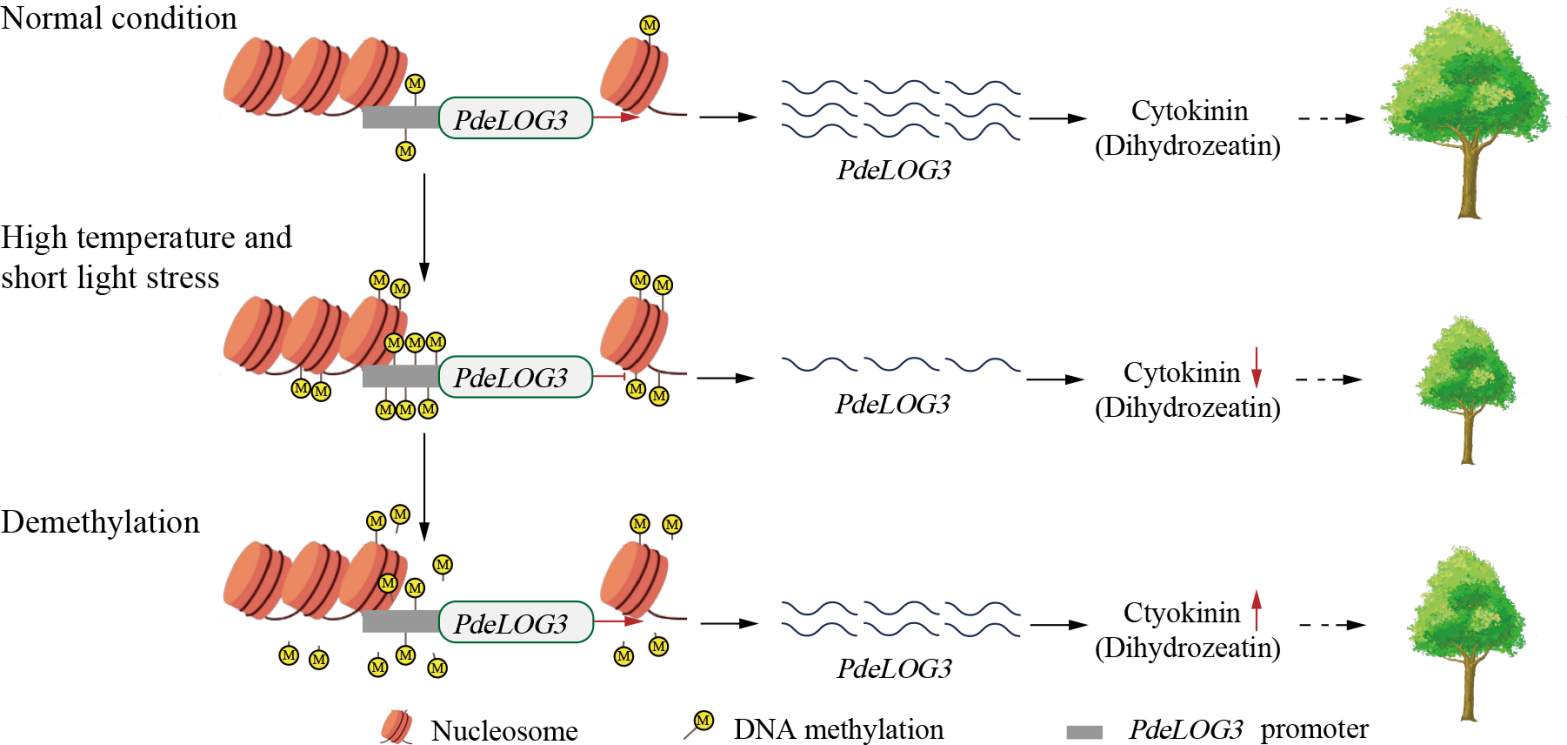
CHH-type hypermethylation in the promoter of *PdeLOG3* may be associated with the general response of *P. deltoides* to HS stress. A proposed model suggests a candidate epigenetic regulatory module: under HS conditions, CHH-type hypermethylation occurs in the promoter region of *PdeLOG3*, suppressing its transcription and thereby reducing cytokinin synthesis and plant growth. Treatment with 5-AzaC reverses this repression through demethylation, restoring *PdeLOG3* expression and cytokinin synthesis, and thereby enhancing adaptability to HS stress.

In summary, our results suggest that HS stress induces CHH hypermethylation in the *PdeLOG3* promoter. This epigenetic modification may suppress *PdeLOG3* gene expression, reduce active cytokinin (dihydrozeatin) synthesis, and consequently inhibit growth. The demethylating agent 5-AzaC can partially reverse this repression, which may enhance the plant's antioxidant capacity and consequently may improve its acclimation to stress (Fig. 7). Thus, CHH-type methylation of the *PdeLOG3* promoter may be involved in the adaptive response to HS stress in *P. deltoides*.

## Discussion

DNA methylation, a key epigenetic modification, has been widely shown to regulate plant responses to diverse environmental stresses, including drought, temperature, light, salinity, and metal toxicity^[7,8,10−12]^. Unlike single genetic variations, the combination of epigenetic and genetic variation under natural selection accelerates the adaptive evolution of plant populations to local environments^[34,35]^. Consistent with previous studies^[6,17]^, the present findings suggest that temperature and light may be among the major environmental differences between temperate and tropical regions, and these factors could play a role as potential barriers to the successful introduction and domestication of temperate-origin poplars in tropical climates (Fig. 1c). Correspondingly, the survival and growth of *P. deltoides* genotypes are markedly reduced when introduced into tropical regions compared with temperate environments (Fig. 1b).

High temperature and short photoperiod frequently elevate DNA methylation levels in plants, with this process also exhibiting sequence-context specificity^[7,8,12,14,34]^. For example, in *A. thaliana*, CHH methylation increases with rising temperature, whereas CG and CHG methylation remain relatively stable^[7]^. We also observed analogous patterns in our analysis of methylation differences between the two regions across various genotypes. In our study, CHH methylation changes were relatively similar among the five *P. deltoides* genotypes. Compared with those in temperate regions, CHH methylation levels in tropical regions increased significantly (average 1.53-fold), with the strongest differences occurring in promoter regions (Figs 2, 3a, b). This contrasts with *A. thaliana*, where temperature-induced CHH hypermethylation occurs primarily in transposons^[3,7,36]^, suggesting that perennial trees may employ unique epigenetic regulatory mechanisms in response to sustained environmental stress. The fact that all five genotypes exhibited similar shifts in CHH-type methylation levels and their variability between the two regions leads us to propose that CHH-type methylation is sensitive to environmental variation and may contribute to the general response of *P. deltoides* to the HS environment in tropical regions.

DNA methylation influences plant growth and environmental adaptability by regulating the transcriptional expression of target genes, and its effects on transcription vary with species, developmental stage, sequence context, genomic region, and target gene^[3,37,38]^. For example, in *O. sativa* exposed to heat and drought, methylation levels were predominantly positively correlated with the expression of stress-responsive genes^[37]^, whereas in *Solanum lycopersicum* during cold storage, no clear correlation was observed between methylation and gene expression^[39]^. In *Gossypium hirsutum*, gene expression under heat stress was negatively correlated with promoter methylation but positively correlated with gene body methylation^[38]^. Consistent with the previous findings, among the 56 shared CHH-DMDGs identified across the five genotype–location comparisons in our study, the numbers of positively and negatively regulated genes were comparable, with the differences attributable mainly to the biological processes involved (Supplementary Table S6; Fig. 5b). Specifically, genes associated with early signaling (*PdeCNGC13*), heat and light stress (*PdeHSP15.7*, *PdeHSFA6b*), and ABA metabolism (*PdeCYP707A4*) exhibited high expression, whereas genes related to auxin (*PdeARF6*), cytokinin (*PdeLOG3*), and cell cycle regulation (*PdeCYCB2*, *PdeNACK2*) showed low expression. Interestingly, these genes all exhibited highly consistent hypermethylation in their promoter regions (Fig. 5c), suggesting that CHH-type DNA methylation in promoter regions may contribute to the growth divergence of *P. deltoides* between the two regions, potentially in association with temperature and light conditions.

Our study also revealed that the five genotypes exhibited marked differences in their sensitivity to the stressful environment of the HK region, and we speculate that this differential sensitivity may be associated with CG and CHG methylation (Figs 1d, 3). Studies in *O. sativa* and *Brassica napus* have shown that heat-sensitive genotypes exhibit stronger increases in methylation under heat stress, but lower methylation levels under normal conditions^[14]^. Consistent with this, PD5 displayed the lowest CG and CHG methylation levels among the five *P. deltoides* genotypes in NY, and exhibited genotype-specific CG- and CHG-type methylation variations between the two regions (Figs 2c, 3a, b). Our early regionalization trials revealed that many *P. deltoides* genotypes introduced from QC provenance, the origin of the highly sensitive genotype PD5, failed to survive in the HK region^[17]^. This likely resulted from the sharp differences in latitude and climate between QC and HK, which exceeded the species' suitable introduction range. Traditionally, CG methylation in plants was considered relatively stable, with limited influence from genotype or environment, and thus not a major regulator of gene expression^[40,41]^. However, recent studies have demonstrated that genome-wide CG methylation (GBM) can vary across large geographic gradients, being shaped by environmental conditions at both provenance and planting sites, especially correlated with latitude^[42]^. For example, *A. thaliana* accessions from colder, high-latitude regions exhibit elevated GBM^[7]^. Unlike *A. thaliana*, PD5 introduced from high-latitude regions (QC) showed lower CG and CHG methylation levels in NY compared with the other four genotypes from low-latitude regions (LA) (Fig. 2c). However, the number of CG- and CHG-type DMRs between genotypes in this region was similar (Supplementary Fig. S2a, S2b). In contrast, a different pattern was observed in the lower-latitude regions (HK). There, PD5 exhibited specific and extensive CG and CHG hypermethylation compared with the other genotypes, with these methylation changes targeting genes primarily involved in fundamental biological processes (Supplementary Fig. S2a, S2b; Fig. 3c). These results offer a possible explanation for the failure of QC-provenance *P. deltoides* in tropical regions: the highly sensitive genotype triggers extensive CG and CHG hypermethylation when it exceeds the species' suitable introduction range, which impairs its fundamental physiological activities, thereby inhibiting its growth and even leading to death. We therefore speculate that perennial trees may have evolved distinct methylation strategies during long-term adaptation. Unlike the extensive and consistent changes in CHH-type methylation under environmental variation, CG- and CHG-type methylation generally exhibit higher stability. This stability may serve as a buffering mechanism that helps *P. deltoides* sustain fundamental physiological activities amid environmental fluctuations by maintaining stable expression of basic metabolic genes (Supplementary Fig. S5; Fig. 3). Supporting this, studies in natural *Quercus lobata* populations have linked CG methylation variation to local adaptation and stress-response plasticity^[5]^.

However, the marked cross-environment variation in CG/CHG methylation was observed solely in PD5, while the other genotypes remained stable, implying that this is likely a genotype-specific response to exceeding the suitable range rather than a general strategy. In contrast, the consistent environmental sensitivity of CHH-type methylation across all genotypes suggests it may act as a general adaptive mechanism enabling *P. deltoides* to cope with the stressful HK environment. To test this hypothesis, we exposed different sensitive genotypes to HS (a simulated combined stress of high temperature and short photoperiod, designed to reflect the key environmental shift from NY to HK, rather than a complete reconstruction of the HK field conditions) and HL (simulating extreme high-temperature stress under future climate scenarios) treatments under controlled indoor conditions. The sole stress of short photoperiod was not considered in this study, as in the natural environments of NY and HK, short photoperiod always occurs in conjunction with high temperature or seasonal transition, rather than in isolation. The stress simulation experiment revealed that the growth differences and SSI values of the three *P. deltoides* genotypes under HS and HL stress treatments were highly consistent with the field trial results (Figs 1b, d, 6a, b), supporting the view that high temperature and short photoperiod are likely key environmental factors affecting poplar growth in the HK region.

To validate the candidate key genes identified in our earlier field trials, we treated plants with the DNA methylation inhibitor 5-AzaC, and subsequently identified *PdeLOG3*, *PdeCNGC13*, *PdeARF6*, and *PdeHSP15.7* as potential methylation-regulated effector genes of HS adaptation in *P. deltoides*, which exhibited clear expression changes under HS stress that were partially reversed by 5-AzaC treatment (Fig. 6h; Supplementary Fig. S6a–S6c). Among these, cyclic nucleotide-gated channels (CNGCs) play a critical role in mediating Ca^2+^ influx across the plasma membrane in response to thermal stress^[43,44]^. This influx promotes ROS accumulation and subsequently activates the transcription of HSPs and heat shock transcription factors (HSFs)^[2,44−46]^. In parallel, auxin response factors (ARF6/8) interact with photoreceptors involved in thermomorphogenic inhibition (e.g., CRY1, PhyB), thereby contributing to HS stress responses^[47−49]^. Consistent with this, 5-AzaC application alleviated HS-induced physiological symptoms, including Ca^2+^ influx, oxidative damage (MDA), and growth inhibition, while enhancing antioxidant capacity (POD, proline) and mitigating the decline in auxin and cytokinin levels (Fig. 6a−g). It is worth noting that Ca^2+^ flux was measured only in root tips and not directly examined in leaf tissues. However, a large body of research has demonstrated that Ca^2+^ waves triggered by local stresses such as salt, high temperature, and high light can be systemically propagated throughout the plant via long-distance signaling pathways (e.g., vascular bundles in the stem) in response to environmental stimuli^[50−53]^. In *Arabidopsis*, for example, herbivore attack initiates a glutamate-triggered Ca^2+^ wave that travels from the wounded leaf through the stem vasculature to roots and distal leaves, rapidly activating systemic defenses^[53]^. Therefore, it is possible that the root tip Ca^2+^ influx observed here reflects a distal component of systemic signaling, which might be linked, indirectly, to transcriptional and epigenetic changes in leaves. These results suggest that DNA methylation dynamics under HS stress may be associated with the stress response of *P. deltoides*, potentially involving changes in Ca^2+^ influx and the biosynthesis of auxin and cytokinin.

Cytokinins have been widely demonstrated to act as positive regulators of plant adaptation to heat and light stress, enhancing stress tolerance through pathways such as promoting reactive oxygen species (ROS) scavenging and inducing heat shock protein (HSP) accumulation^[54−56]^. The *LOG3* gene plays a key role in cytokinin biosynthesis by converting cytokinin nucleotides across the plasma membrane into cytokinin nucleobases, particularly dihydrozeatin^[31−33]^. We observed that HL and HS stress induced CHH hypermethylation in a conserved 291-bp promoter fragment of *PdeLOG3*, and this methylation was negatively correlated with both its transcript levels and dihydrozeatin content (Supplementary Fig. S8; Fig. 6i, j). 5-AzaC treatment reduced CHH methylation in the promoter region of *PdeLOG3* and partially restored its expression, which increased cytokinin levels and consequently enhanced antioxidant capacity, ultimately promoting resource reallocation towards stress tolerance (Fig. 6a–g). Thus, *PdeLOG3* may be a potential node in the epigenetic regulation of *P. deltoides* adaptability under HS stress, offering the possibility of establishing an epigenetic link between HS stress adaptation and cytokinin-driven growth regulation. Although the correlative evidence presented is compelling, we acknowledge that the current findings are primarily based on methylation profiling and pharmacological reversal experiments, and direct genetic evidence linking *PdeLOG3* promoter methylation to HS stress adaptation remains lacking. Nonetheless, the linked changes in methylation, expression, and hormone levels observed in this study, along with the partial reversal by 5-AzaC, seem to offer indirect evidence that *PdeLOG3* might function as a potential epigenetic regulator under HS stress. In this context, we note that because 5-AzaC causes genome-wide demethylation and may induce additional physiological effects (e.g., elevated MDA levels under normal growth conditions) (Fig. 6f), the observed recovery of *PdeLOG3* expression and cytokinin content should be interpreted as supportive but not definitive evidence for a promoter-specific methylation mechanism. Future studies employing targeted epigenetic editing (e.g., CRISPR/dCas9-TET to demethylate the specific promoter region) or generating *PdeLOG3* knockout/overexpression transgenic lines will be essential to validate the functional role of this epiallele. Furthermore, investigating whether a similar epigenetic regulatory module operates in other woody perennials would be of interest in understanding the generality of such stress acclimation strategies.

**Figure 8:**
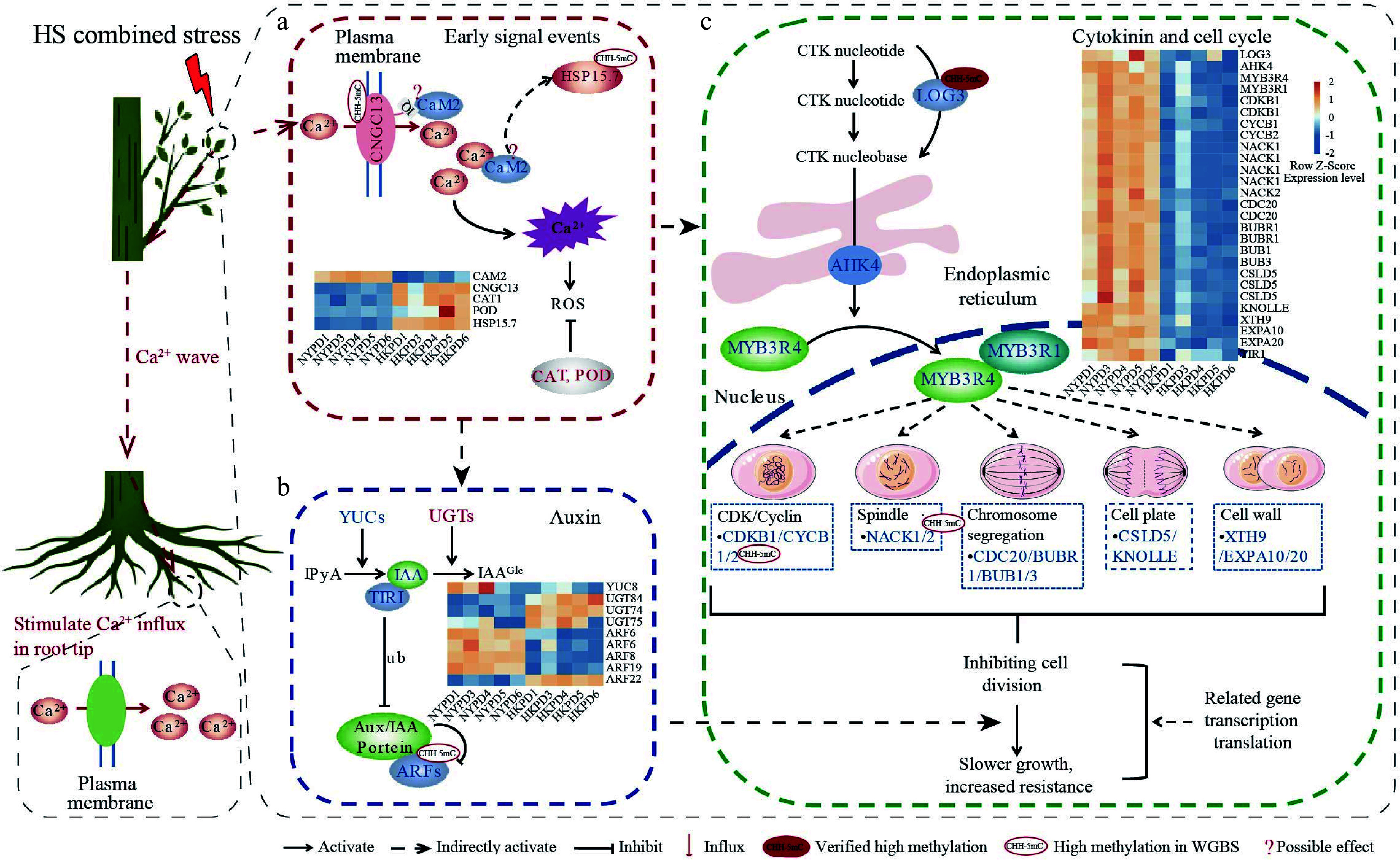
A proposed molecular network of promoter CHH hypermethylation associated with the general response of *P. deltoides* to HK region stress environment. (a) Under HS stress, CHH hypermethylation in the promoter of *PdeCNGC13* enhances its transcription, causing a sharp increase in intracellular Ca^2+^ levels. This triggers large-scale ROS production, which in turn activates CAT and POD to mitigate oxidative damage. Inhibited CaM2 releases its binding to the IQ motif at the C-terminus of *PdeCNGC13*, further activating Ca^2+^ influx channels. The Ca^2+^–CaM signaling system may also induce *PdeHSP15.7* activity under HS stress. It should be noted that Ca^2+^ flux was measured in root tips and may reflect systemic signaling rather than direct changes in leaf tissues. (b) Early signal stimulation suppresses auxin biosynthesis. Inhibited *YUCs* reduce IAA synthesis from the IPyA precursor, while highly expressed *UGTs* glycosylate IAA into IAA^Glc^, collectively lowering auxin levels. Inhibited *TIR1* reduces its binding to IAA and Aux/IAA proteins, resulting in increased Aux/IAA–ARF complexes that block ARF-mediated transcription. (c) Early signal stimulation and reduced auxin biosynthesis jointly suppress cell division and growth. Promoter CHH hypermethylation of *PdeLOG3* represses its transcription, limiting the conversion of cytokinin nucleotides to nucleobases. Cytokinin transport is further reduced by AHK4 in the endoplasmic reticulum, decreasing active cytokinin levels. Short photoperiod stress suppresses the nuclear translocation of *MYB3R4*, while reduced activity of *MYB3R1* and *MYB3R4* diminishes downstream regulation of cell cycle genes (*CDKB1*, *CYCB1*/2), spindle formation (*NACK1/2*), chromosome segregation (*CDC20*, *BUBR1*, *BUB1/3*), cell plate formation (*CSLD5*, *KNOLLE*), and cell wall formation (*XTH9*, *EXPA10/20*), thereby inhibiting cell division and growth. Abbreviations: CNGC13, Cyclic nucleotide-gated ion channel 13; CaM2, Calmodulin-2; HSP15.7, Heat Shock Protein 15.7; YUCs, YUCCA; UGTs, UDP-glucosyltransferase; TIR1, Transport Inhibitor Response 1; IPyA, Indole-3-pyruvic acid; IAA, Indole-3-acetic acid; IAA^Glc^, Indole-3-acetic acid-β-D-glucoside; LOG3, Lonely Guy 3; AHK4, Arabidopsis histidine kinase 4; MYB3R1, MYB domain protein 3R1; MYB3R4, MYB domain protein 3R4; CDKB1, Cyclin-dependent kinase B1; CYCB1/2, Cyclin B1/B2; NACK1/2, Kinesin-like protein NACK1/2; CDC20, Cell division cycle 20; BUBR1, Mitotic spindle checkpoint protein BUBR1; BUB1/3, Mitotic checkpoint serine/threonine kinases BUB1/3; CSLD5, Cellulose synthase-like D5; XTH9, Xyloglucan endotransglucosylase/hydrolase 9; EXPA10/20, Expansin A10/A20; ub, ubiquitination.

Plant adaptation to environmental change operates through a complex and coordinated molecular response network. Genes influenced by DNA methylation are known to be involved in the differential expression of numerous downstream targets, thereby contributing to physiological and biochemical responses. Based on the integrated results of WGBS, RNA-seq, and validation experiments, we preliminarily constructed a potential molecular response framework for DNA methylation–associated acclimation of *P. deltoides* to the stress environment in the HK region (Fig. 8). In this model: (i) HK region stress environment is associated with widespread CHH-type hypermethylation; (ii) these epigenetic changes correlate with the activation of stress-response pathways (e.g., CNGC13-mediated Ca^2+^ signaling), while potentially being associated with the suppression of growth pathways via putative genes such as *PdeLOG3* (cytokinin synthesis) and *PdeARF6* (auxin signaling); and (iii) in sensitive genotypes like PD5, a provenance-environment mismatch may trigger additional CG/CHG hypermethylation in genes essential for basal metabolism, potentially leading to energy depletion and growth inhibition.

## Conclusions

Based on five *P. deltoides* genotypes across two field sites and complementary controlled-environment chamber experiments, our study suggests that CG/CHG and CHH methylation may play distinct roles in environmental stress responses. Specifically, within the scope of this study, stable CG/CHG methylation may serve as a potential feature of environmental adaptation of *P. deltoides*. In contrast, labile CHH methylation may function as a potential epigenetic regulator, fine-tuning stress-responsive adaptation and offering some potential for breeding trees resilient to climate change. We identified *PdeCNGC13*, *PdeARF6*, *PdeLOG3* and *PdeHSP15.7* as potential methylation-associated effector genes that may be involved in HS adaptation. Furthermore, the possible regulatory mechanism of *PdeLOG3* was delineated, suggesting a candidate epigenetic module between HS stress adaptation and cytokinin-driven growth regulation. Collectively, our work preliminarily uncovers the molecular mechanisms underlying DNA methylation-associated environmental adaptation in poplar and provides potential genetic targets for breeding climate-resilient trees.

## SUPPLEMENTARY DATA

Supplementary data to this article can be found online.

## Data Availability

The datasets generated during and/or analyzed during the current study are available from the corresponding author on reasonable request. The original RNA-sequencing and WGBS data have been deposited into the Genome Sequence Archive at National Genomics Data Center, China National Center for Bioinformation/Beijing Institute of Genomics, Chinese Academy of Sciences (GSA: CRA016127 and CRA031106), which is publicly accessible at https://ngdc.cncb.ac.cn/gsa.
