# Supplementary material for: Molecular regulatory mechanisms of Populus deltoides under combined high temperature and short-photoperiod stress associated with DNA methylation
Source: For Res (Fayettev). 2026 Jul 31;6:e025. doi: 10.48130/forres-0026-0025 (PMC13447283; doi:10.48130/forres-0026-0025)
Supplement: Supplementary file 1 [file FR-2026-6-0025-S1.zip › 10.48130_forres-0026-0025-Suppl-FigureS2.pdf]

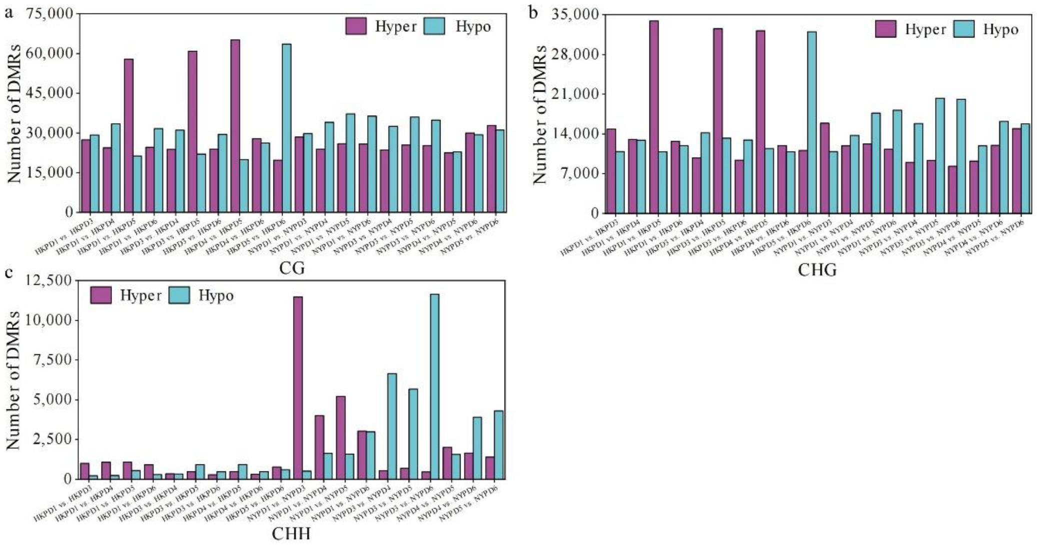

Fig. S2 Count of DMRs in genotype-genotype comparisons within each location.The numbers of CG-DMRs (a), CHG-DMRs (b), and CHH-DMRs (c) in genotype–genotype comparison groups in HK and NY regions.
