# Supplementary material for: Molecular regulatory mechanisms of Populus deltoides under combined high temperature and short-photoperiod stress associated with DNA methylation
Source: For Res (Fayettev). 2026 Jul 31;6:e025. doi: 10.48130/forres-0026-0025 (PMC13447283; doi:10.48130/forres-0026-0025)
Supplement: Supplementary file 1 [file FR-2026-6-0025-S1.zip › 10.48130_forres-0026-0025-Suppl-FigureS4.pdf]

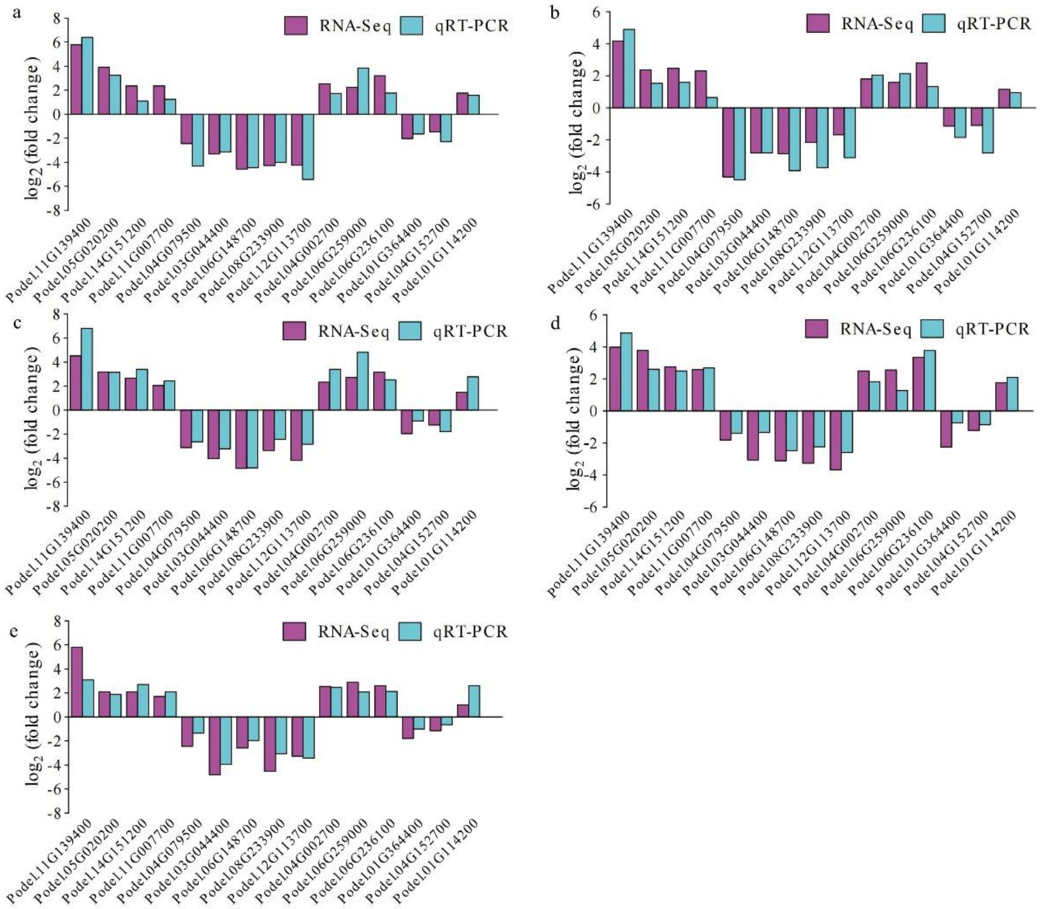

Fig. S4 Differential expression of 15 genes validated by RNA-seq and qRT-PCR.(a) HKPD1 vs. NYPD1. (b) HKPD3 vs. NYPD3. (c) HKPD4 vs. NYPD4. (d) HKPD5 vs. NYPD5. (e) HKPD6 vs. NYPD6.
