# Supplementary material for: Molecular regulatory mechanisms of Populus deltoides under combined high temperature and short-photoperiod stress associated with DNA methylation
Source: For Res (Fayettev). 2026 Jul 31;6:e025. doi: 10.48130/forres-0026-0025 (PMC13447283; doi:10.48130/forres-0026-0025)
Supplement: Supplementary file 1 [file FR-2026-6-0025-S1.zip › 10.48130_forres-0026-0025-Suppl-FigureS6.pdf]

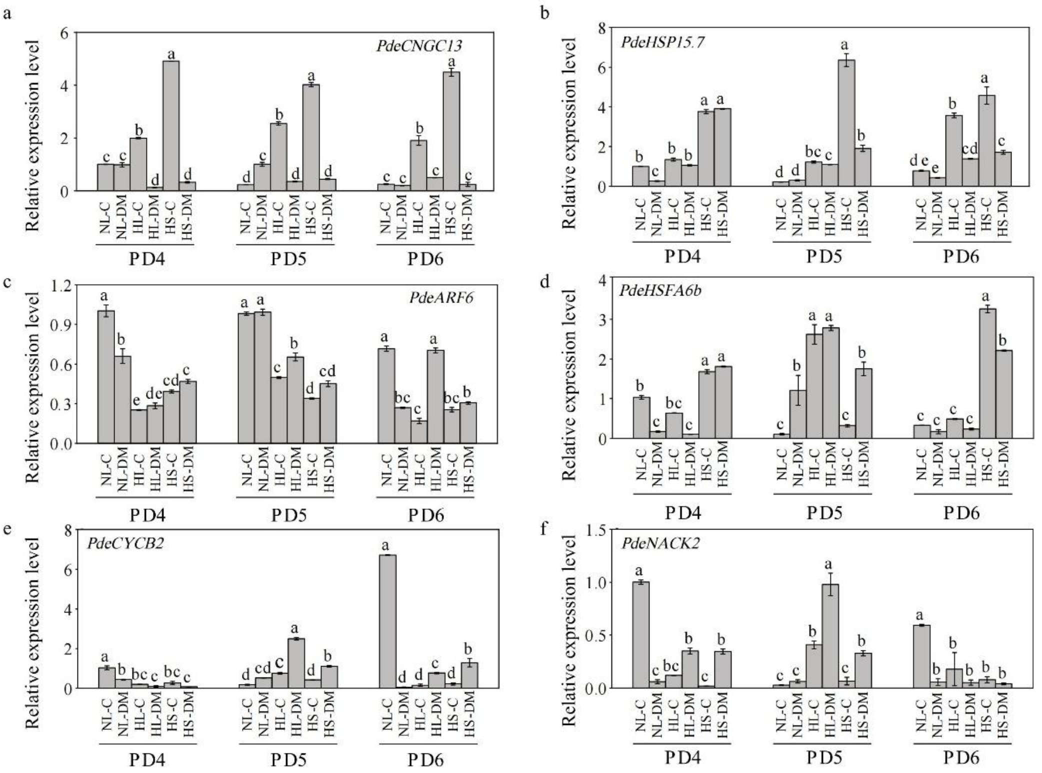

Fig. S6 Differential expression of key genes in *P. deltoides* with and without 5-AzaC under NL, HL, and HS conditions.Relative expression levels of *PdeCNGC13* (a), *PdeHSP15.7* (b), *PdeARF6* (c), *PdeHSFA6b* (d), *PdeCYCB2* (e), and *PdeNACK2* (f) after 7 days of stress. Multiple comparisons were performed using the Tukey test (n = 4), with different letters indicating significant differences ($P < 0.05$). Error bars represent ± SD. NL, normal temperature with long light; HL, high temperature with long light; HS, high temperature with short light; C, without 5-AzaC; DM, with 5-AzaC.
