# Supplementary material for: Molecular regulatory mechanisms of Populus deltoides under combined high temperature and short-photoperiod stress associated with DNA methylation
Source: For Res (Fayettev). 2026 Jul 31;6:e025. doi: 10.48130/forres-0026-0025 (PMC13447283; doi:10.48130/forres-0026-0025)
Supplement: Supplementary file 1 [file FR-2026-6-0025-S1.zip › 10.48130_forres-0026-0025-Suppl-FigureS7.pdf]

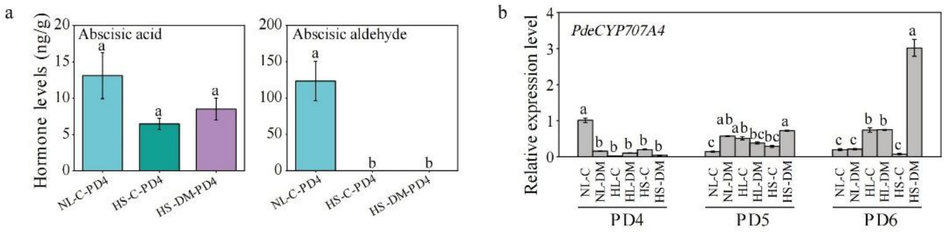

Fig. S7 Differences in abscisic acid content and expression of related genes in *P. deltoides* with and without 5-AzaC under NL, HL, and HS conditions.(a) Abscisic acid content after 7 days of stress treatment.(b) Relative expression level of *PdeCYP707A4*gene after 7 days of stress treatment.
