# Supplementary material for: Molecular regulatory mechanisms of Populus deltoides under combined high temperature and short-photoperiod stress associated with DNA methylation
Source: For Res (Fayettev). 2026 Jul 31;6:e025. doi: 10.48130/forres-0026-0025 (PMC13447283; doi:10.48130/forres-0026-0025)
Supplement: Supplementary file 1 [file FR-2026-6-0025-S1.zip › 10.48130_forres-0026-0025-Suppl-FigureS8.pdf]

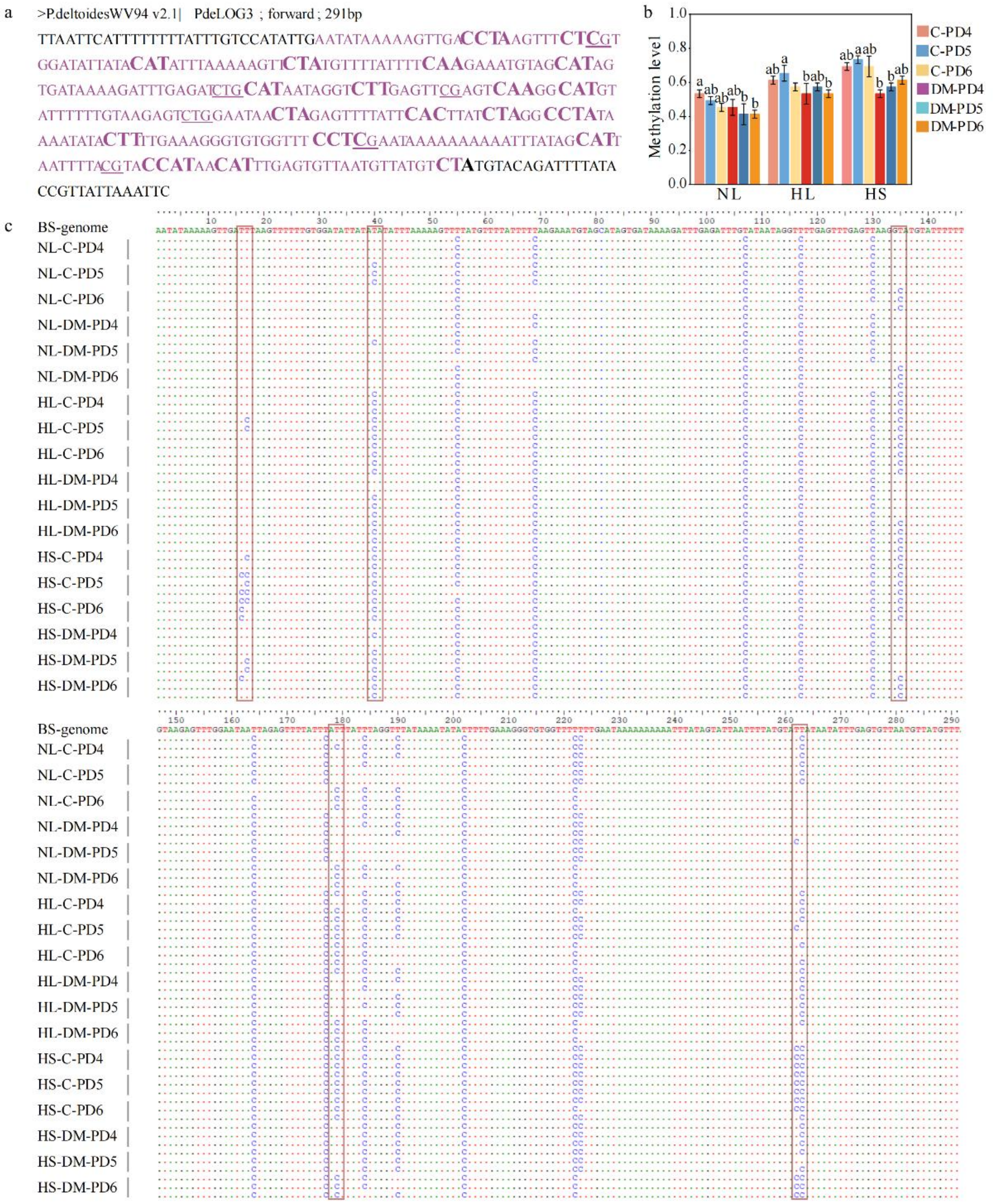

Fig. S8 Genomic distribution and methylation analysis of the *PdeLOG3* gene in *P. deltoides*.(a) Distribution of CG, CHG, and CHH C sites in the promoter region of the *PdeLOG3* gene in the *P. deltoides*reference genome.The target fragment (291bp, shown in purple font) along with its 30 bp upstream and downstream sequence is displayed. A total of 31 C sites were identified, including four CG sites, two CHG sites, and 25 CHH sites. (b) Average methylation level of C sites in the target fragment (291bp).Different lowercase letters indicate significant differences ($P< 0.05$) among genotypes/treatments within the same growth condition (Tukey test, n = 3). (c) Landscape of mCHH methylation sites in each sample detected by BS-PCR.
